# Impact of federal funding for graduate medical education on residency program size: Evidence from the Affordable Care Act

**DOI:** 10.1371/journal.pone.0318626

**Published:** 2025-02-10

**Authors:** Cici McNamara, Tehreem Hussain

**Affiliations:** 1 School of Economics, Georgia Institute of Technology, Atlanta, Georgia, United States of America; 2 Rollins School of Public Health, Emory University, Atlanta, Georgia, United States of America; Universitas 17 Agustus 1945 - Jakarta, INDONESIA

## Abstract

Primary care and rural physician shortages are a present and growing concern to policy makers. We assessed three Affordable Care Act (ACA) provisions that changed the maximum number of residents teaching hospitals could be reimbursed for, an element of graduate medical education (GME) funding known as the resident cap. The results show that an increase in a hospital’s resident cap of one slot under one of these ACA provisions in 2010 is associated with an increase in residency program size of approximately one full-time equivalent resident. We find important heterogeneity in the magnitude of the association between resident cap changes and program growth across ACA provisions, as well as in whether these associations are driven by changes in primary or non-primary care program growth. These results suggest that targeted changes to GME funding may be an effective tool in helping address physician shortages.

## Introduction

The U.S. faces a projected physician shortage of between 37,800 and 124,000 by the year 2024 [1]. Burnout and an aging workforce have increased physician turnover in recent years, while insurance expansions and an aging patient population have increased healthcare demand [2–4]. Concerns about a shortage have only been exacerbated by the COVID-19 pandemic, which increased turnover rates across nearly all segments of the healthcare workforce [5]. Of particular concern to policymakers are physician shortages in primary care specialties, rural areas, and the intersection of the two. Over 100 million people in the U.S. live in a primary care Health Professional Shortage Area (HPSA) and 62 percent of primary care HPSAs are in rural areas [6, 7]. It is important to identify policies that are effective at addressing these shortages given that primary care physician supply is negatively associated with mortality, hospitalizations, and rates of late-stage cancer diagnosis [8–11].

Policy solutions for addressing the physician shortage in the U.S. have historically included expansions of medical student loan support through the Retirement Parity for Student Loans Act, along with facilitating the integration of international medical graduates through the Conrad State 30 and the Physician Access Reauthorization Acts [12]. However, physician workforce shortages are not unique to the U.S.: the National Health Service (NHS) in the United Kingdom recently published the NHS Long Term Workforce Plan that focuses on investing in medical training, and aims to grow medical school class sizes by 60–100% by 2031 [13]. Similarly, in Australia, the World Health Organization has predicted a coverage gap of 10 million healthcare workers by 2030, prompting the government to publish the National Medical Workforce Strategy to prioritize the training of general practitioners and practice efficient resource allocation in regards to distribution of doctors [14].

Another proposed remedy for the primary care and rural physician shortages in the U.S. is an increase in funding for graduate medical education provided to rural teaching hospitals and those with primary care residencies [15]. Graduate medical education (GME) is the umbrella designation for the training that physicians receive following graduation from medical school. During this time, trainees specialize or subspecialize as residents or fellows [16]. The federal government reimburses teaching hospitals for training residents through Medicare payments. Medicare payments constitute the vast majority of funding for GME and totaled $16.2 billion in 2020. Medicare reimbursements are made for both the direct and indirect costs of graduate medical education. Direct Graduate Medical Education (DGME) payments are meant to cover direct costs associated with operating a residency program such as salaries and administrative costs, whereas Indirect Graduate Medical Education (IME) payments are intended to reimburse hospitals for the cost of relatively inefficient care provided by trainees in comparison to their attending physician counterparts [17].

Teaching hospitals are subject to caps on the number of residents they can be reimbursed for training. Hospitals’ reimbursements for GME are a function of the number of residents they train up to a cap, at which point reimbursements are a function of the cap and do not increase with program size. These caps were implemented as part of the Balanced Budget Act of 1997 and typically correspond to the number of residents the hospital was training in 1996 [18]. Most teaching hospitals therefore train more residents than they are reimbursed for and may face financial barriers to residency program expansion which funding increases could help alleviate.

Sections 5503 and 5506 of the Affordable Care Act (ACA) provided for changes in the GME funding of certain hospitals. In both cases, funding changes were implemented through changes in treated hospitals’ resident caps. Hospitals were eligible to receive cap increases for training additional primary residents under Section 5503 if they were located in rural or high-need areas. Cap increases under Section 5503 were allocated from cap decreases implemented under the same provision. Hospitals were subject to cap decreases under Section 5503 if the number of residents they trained was below their cap, regardless of the hospital’s location. These hospitals had their caps reduced by 65 percent of the difference between their resident caps and counts, and the aggregate reduction in resident counts served as the set of available slots for awardees of Section 5503 cap increases. Hospitals that received residency cap increases under Section 5503 were required to use at least 75 percent of those additional slots to expand the size of their primary care or general surgery residency programs and could not be used to fund already enrolled residents. Changes to GME payments to hospitals participating in Section 5503 went into effect on July 1, 2011.

Section 5506 allowed for the resident caps of teaching hospitals that closed to be redistributed to a nearby hospital, whereas those funded residency slots were previously lost along with the closed hospital. These changes went into effect for all hospitals that closed on or after March 23, 2008, beginning on February 28, 2012 [19]. Hospital closures are particularly common in rural areas: there were 136 rural hospital closures between 2010 and 2020 [20]. The preservation of closed teaching hospitals’ residency funding is therefore important for sustaining residency training programs in rural areas. Whether the increase in funded residency slots implemented under Sections 5503 and 5506 resulted in an increase in the residency program size of participating hospitals depends in part on the elasticity of resident labor supply to those hospitals. Given that many of these hospitals were located in rural and high need areas, it is possible that labor supply to those hospitals was relatively inelastic, which would result in fewer than one additional resident recruited per additional slot.

There has been continued interest in using federal residency funding to affect the number of residents trained in primary care and rural areas since the implementation of Sections 5503 and 5506 over a decade ago. Most recently, Section 126 of the Consolidated Appropriations Act made available more than 1,000 residency slots to qualifying hospitals under criteria similar to those used in the ACA provisions [21]. At the same time, there is little consensus on the relationship between GME funding and the quality and quantity of GME that hospitals provide [22, 23]. We sought to develop a better understanding of the effectiveness of residency funding changes at increasing the number of residents trained in targeted specialties and geographies. We did so by characterizing the hospitals who received funding changes under Sections 5503 and 5506 of the ACA and estimating the associations between these funding changes and growth in residency program size.

## Materials and methods

We obtained data on hospitals’ resident counts and GME payments for the years 2007 and 2013 from the Centers for Medicare and Medicaid Service’s Healthcare Cost Report Information Systems (HCRIS) hospital cost reports [24, 25]. This timeframe provides a six-year symmetric window around the passage of the ACA and precedes the implementation of the ACA’s Medicaid expansion, which may have also influenced hospitals’ employment decisions and cofounded our results. It also accounts for Medicare reimbursements being a function of the lesser of the residency cap and three-year rolling average of the hospital’s resident count. We restricted our sample to teaching hospitals, which we define as those that indicate involvement in training residents in approved GME programs in 2007 or that receive GME funding revisions under Sections 5506 or 5503 of the Affordable Care Act. There were 1,288 hospitals that satisfied these criteria, of which 1,216 (94%) were also present in the cost report in 2013.

The HCRIS contains information on the number of resident full-time equivalents (FTEs) that each teaching hospital trained, resident caps, and Medicare GME payments. Each of these variables is reported separately for DGME and IME, as the calculation of FTEs and payments differs for these two types of costs [26]. Variables are reported and payments made based on resident counts across all of the allopathic and osteopathic programs that the hospital provides training for and are not broken out by specialty. Hospitals do report DGME FTE counts separately for residents enrolled in primary care and non-primary care programs.

Hospitals were eligible to apply for a cap increase under Section 5503 if they were located in a state in the bottom quartile of the 2009 resident-to-population ratio distribution, a state in the top ten of the 2009 primary care HPSA-to-population ratio distribution, or a rural area. Rural areas were defined as counties outside of a Core Based Statistical Area (CBSA). Hospitals that satisfied any of these eligibility criteria could apply for a residency cap increase under Section 5503. Section 5503 specified that 70 percent of available slots were to be allocated to hospitals eligible under the resident-to-population ratio criteria, while the remaining 30 percent were to go to hospitals that qualified under the primary care HPSA-to-population ratio or rural criteria. Applications for the 70 percent pool were considered in ascending order based on the resident-to-population ratio of the hospital’s state, while applications for the 30 percent pool were considered in descending order based on the primary-care HPSA-to-population ratio of the hospital’s state. Using data on state’s 2009 resident-to-population and primary care HPSA-to-population ratios from policy documents on these ACA provisions and geocodes contained in the HCRIS, we constructed variables indicating each hospital’s eligibility for a cap increase under Section 5503 and the source of that eligibility [27].

We used publicly available data from CMS on residency cap changes implemented under Sections 5503 and 5506 to construct residency cap change measures. Residency cap changes made under Section 5503 constituted a one-time reallocation of funded residency slots across hospitals that went into effect beginning with cost reporting periods occurring on or after July 1, 2011. Unlike Section 5503, which was a one-time reallocation, Section 5506 residency slot reallocations have been implemented in multiple rounds since the ACA’s passage to address the most recent hospital closure activity. In constructing the Section 5506 residency cap increase measure, we focus on the first round of reallocations, which applied to hospital closures that occurred between March 23, 2008 and August 3, 2010.

We estimated the following equation by ordinary least squares:

$${\Delta NumRes}_{h}=\alpha +\beta \Delta {ResCap}_{h}+\mu_{s(h)}+\varepsilon_{h}$$

Here, Δ*NumRes_h_* is the change in hospital *h*’s number of full-time equivalent (FTE) residents between the years 2013 and 2007: ${\Delta NumRes}_{h}={NumRes}_{h,t=2013}-{NumRes}_{h,t=2007}.\;\Delta {ResCap}_{h}$ is the change in hospital *h*’s residency funding cap implemented under Sections 5506 or 5503. *μ*_*s(h)*_ are fixed effects for the state *s* in which hospital *h* is located. Two teaching hospitals that are the only teaching hospitals in their respective states are grouped together under a single fixed effect.

We supplemented our hospital-level data with county-level data on general practice physicians per ten thousand persons from the Area Health Resource File (AHRF). Hospitals in Puerto Rico were excluded from analyses of provider density as the AHRF does not contain data from U.S. territories.

## Results

Our first aim was to describe the baseline characteristics of teaching hospitals that received residency cap increases under ACA provisions and how they compare to teaching hospitals that did not receive cap increases. We also examined how the geographic characteristics relevant for determining eligibility for a cap increase under Section 5503 compare across hospitals that did and did not receive a cap increase under the provision. More detailed summary statistics for our full sample of teaching hospitals active in 2007 can be found in the online Supplement.

Our second aim was to estimate the association between residency cap changes implemented as part of the ACA and changes in resident counts. We compute the change in resident counts for each hospital as the difference between its 2013 number of residents and its 2007 number of residents. We compute this difference for four measures of resident counts–all DGME, primary care DGME, non-primary care DGME, and all IME–and regress these measures on measures of residency cap changes implemented as part of the ACA. We construct and run regressions using as our independent variable of interest one of four residency cap change measures: the increase from Section 5506, the increase from Section 5503, the decrease from Section 5503, and the cumulative change across the previous three measures. This measure will equal zero for teaching hospitals that were not treated under either Sections 5506 or 5503. We include all teaching hospitals in specifications using the cumulative cap change as the independent variable of interest. For specifications using provision-specific cap change measures, we include in our estimation sample only hospitals treated under that provision or treated under no provision in order to account for the provision-specific stipulations discussed above. We include state fixed effects in all regressions to control for the effect of state policies on residency program growth. All regressions are weighted by 2007 hospital discharge volumes. The full regression specification results can be found in the online Supplement.

**Fig 1:**
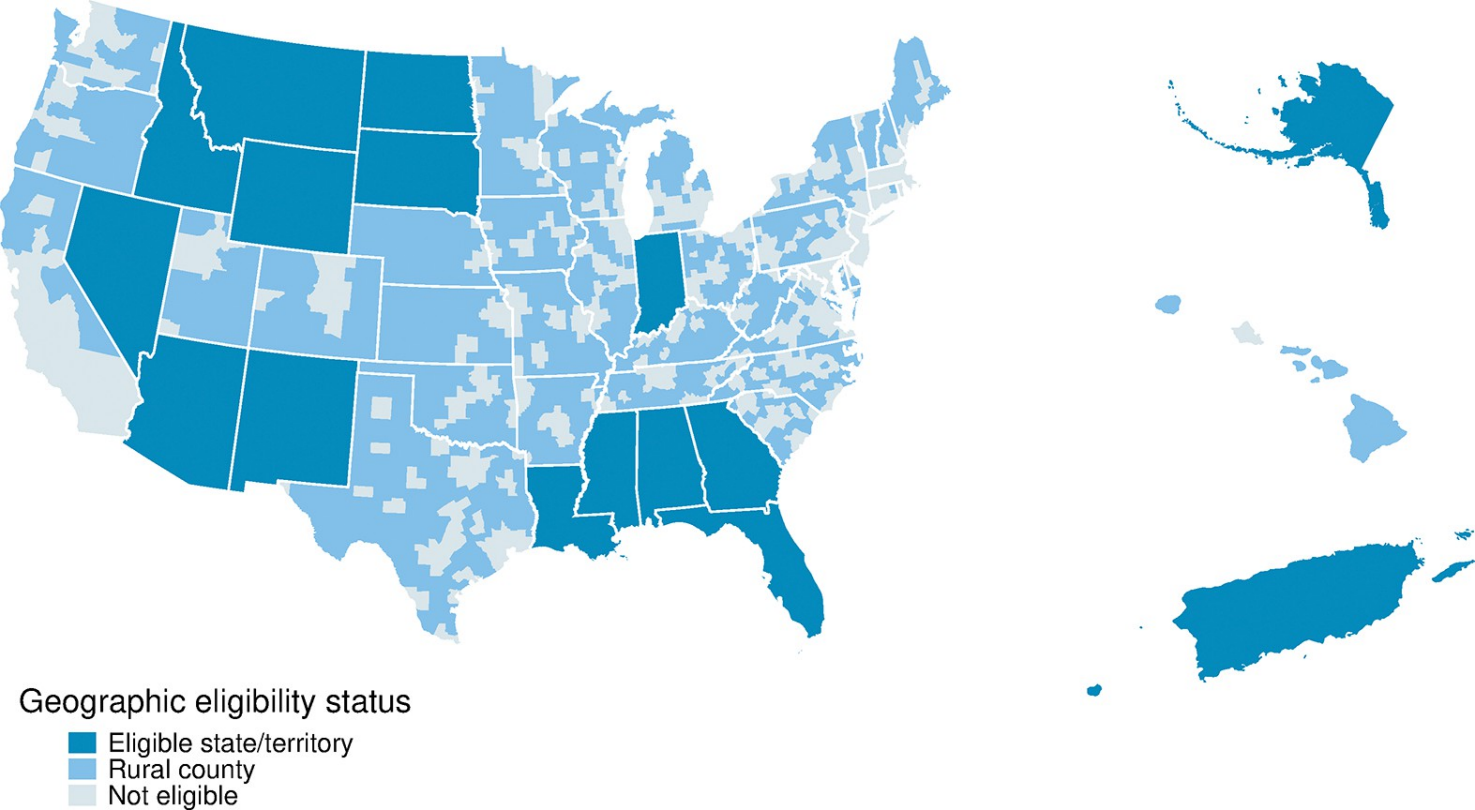
Map of eligibility criteria for Section 5503 cap increases, 2011. Source: Authors’ analysis of data on the distribution of additional residency positions under Section 5503 of the ACA from the Centers for Medicare & Medicaid Services, 2011. Notes: Eligible states were those in the bottom quartile of the resident-to-population distribution or the top ten of the primary care HPSA-to-population ratio distributions. Under Section 412.62(f)(ii), rural counties are those outside of Core Based Statistical Areas not including Litchfield County, Connecticut; York County, Maine; Sagadahoc County, Maine; Merrimack County, New Hampshire; and Newport County, Rhode Island.

Eligibility criteria for a residency cap increase under Section 5503 were a function of state-level measures of need and the rural status of the hospital’s county. Fig 1 illustrates the criteria under which hospitals would (fail to) qualify for a Section 5503 increase based on their location. Locations in dark blue are in high-need states. Locations in light blue are located outside of a CBSA and are therefore rural. Of the fourteen qualifying states in dark blue, five had no teaching hospitals that receive a Section 5503 cap increase: Arizona, Georgia, North Dakota, South Dakota, and Wyoming. While there were hospitals in the qualifying states of Arizona and Georgia that applied for Section 5503 cap increases, none received one as the pool of slots to be reallocated had been exhausted by the time applications of hospitals from these states were considered. Of the hospitals that received Section 5503 cap increases, 39 (67%) qualified via their state’s resident-to-population ratio, 14 (24%) via their state’s primary care HPSA-to-population ratio, and 5 (9%) via being located in a rural county. 23 hospitals in the southeast received Section 5503 cap increases, the most of any region. 17 of these 23 were located in Florida.

Table 1 summarizes the baseline (as of 2007) characteristics of teaching hospitals, stratified by whether and under what ACA provision they received a cap increase. The average residency cap change for both DGME and IME was 0.5. Of the 1,288 teaching hospitals active in 2007, 50 received cap increases under Section 5506. The average size of both the DGME and IME resident cap increases for hospitals treated under Section 5506 was comparable to the increase implemented under Section 5503. Resident cap increases implemented under Section 5503 were larger and concentrated among fewer (58) hospitals than the cap decreases implemented under the same provision, which were taken from 257 unique hospitals. Hospitals that received cap decreases under Section 5503 differed from the average hospital and their counterparts that received cap increases in that they on average had resident counts below their caps. Relative to the full sample, hospitals that received Section 5506 cap increases were in counties with lower general practice physician supply. Lastly, we note that there is not a perfect correspondence between the ranking of the samples in Table 1 by average baseline resident cap size and by average baseline GME payment amount. This discordance stems from the fact that while GME reimbursements are weakly increasing functions of the residency cap, they are also parameterized by hospital characteristics including but not limited number of beds and Medicare patient load.

**Table 1:** Summary of teaching hospital characteristics and graduate medical education funding, 2007.

	All teaching hospitals	Section 5506 hospitals	Section 5503 hospitals	
			Increase	Decrease
Number of treated hospitals	1,288	50	58	257
General practice physicians per ten thousand	3.3	2.4	4.0	3.2
Annual discharges (thous.)	15.4	29.5	21.8	12.4
Size of resident cap change				
DGME	0.5	12.0	12.5	-2.7
IME	0.5	11.4	10.8	-2.3
Baseline resident cap				
DGME	68.2	216.8	73.8	42.7
IME	61.4	199.7	64.1	40.2
Baseline number of residents trained				
DGME	73.2	237.3	80.5	36.7
IME	66.3	222.2	72.5	35.1
Baseline GME payment (million USD)				
DGME	2.3	8.7	1.9	1.4
IME	4.6	17.4	3.8	2.4

Source: Author’s analysis of data on GME funding and resident training from CMS’ Hospital Cost Report Information System (HCRIS) and of area-level general practice physician supply from the Area Health Resource File (AHRF). Notes: Data on baseline characteristics corresponds to 2007. Cells contain means. The resident cap change measure for all teaching hospitals is the cumulative change across Sections 5506 and 5503, while the resident cap change for the provision-specific samples corresponds to the change from the corresponding provision alone. General practice physicians per thousand is computed at the county-level. Statistics for area-level physician supply do not include observations from Puerto Rico.

Table 2 summarizes the associations between changes in residency program size and cap changes implemented as part of the ACA. Column (1) provides estimates for regression specifications estimated on the full balanced panel using the cumulative resident cap change as the independent variable. The results show a correlation between cumulative cap changes and resident counts of nearly one for both DGME and IME resident counts. When this correlation is decomposed into primary and non-primary care DGME resident counts, we see that the majority of this correlation is driven by the association between cap changes and the number of non-primary care residents utilized.

**Table 2:** Association between changes in residency funding caps and number of residents trained, 2007 to 2013.

	(1)	(2)	(3)	(4)
	Cumulative resident cap change	Section 5506 change	Section 5503 increase	Section 5503 decrease
DGME residents	0.98***	1.31**	0.75***	0.19
DGME primary care residents	0.27**	0.27	0.33**	-0.08
DGME non-primary care residents	0.61***	0.86**	0.37*	0.25
IME residents	0.99***	1.31**	0.79***	0.12
Number of observations	1,216	913	921	1,110

Source: Author’s analysis of data on GME funding and resident training from CMS’ Hospital Cost Report Information System (HCRIS). Notes: Each cell contains the estimated coefficient of an ordinary least squares regression of the row’s dependent variable on the column’s independent variable and state fixed effects. Regressions in column (1) were performed on the sample of all teaching hospitals in the balanced panel. Regressions in column (2) were performed on the subsample of teaching hospitals that participated in Section 5506 and those that did receive any type of cap change. Regressions in column (3) were performed on the subsample of teaching hospitals that received a cap increase under Section 5503 and those that did receive any type of cap change. Regressions in column (4) were performed on the subsample of teaching hospitals that received a cap decrease under Section 5503 and those that did receive any type of cap change. **p < 0.05 ***p < 0.01 ****p < 0.001

Columns (2) through (4) provide estimates for regression specifications that estimate the associations between provision-specific changes in resident caps and resident counts. Column (2) shows that a one slot increase in a hospital’s resident cap under Section 5506 is associated with an increase in both DMGE and IME resident counts of approximately 1.3. This correlation is driven by an increase in non-primary care resident counts; there is no statistically significant relationship between Section 5506 increases and primary care resident counts.

Column (3) shows that a one slot increase in a hospital’s resident cap under Section 5503 is associated with an increase in DGME resident counts of 0.75 FTEs and in IME resident counts of 0.79 FTEs. Results for specialty-specific DGME counts show that this result is driven almost equally by increases in primary and non-primary resident counts. Results for Section 5503 decreases are provided in column (4) and show no correlation with any resident count measure.

## Discussion

Our examination of the association between medical resident funding cap changes implemented under the ACA and residency program growth shows that increases in graduate medical education funding are positively associated with changes in program size. We note that the magnitude and source of program growth among hospitals that received resident cap increases differed for the two provisions. Section 5503 cap increases were associated with residency program growth that only just constituted compliance with the provision’s stipulation that at least 75 percent of cap increases be used to fund the expansion of primary care or general surgery residency programs. Section 5506 increases, which were implemented at hospitals with relatively larger residency programs located in areas where residency slot supply for medical school graduates had recently declined, had meaningfully larger associations with program growth. These results highlight the importance of policy design as it relates to the eligibility criteria for receiving and keeping GME funding increases and may be consistent with smaller programs facing relatively higher costs from scaling up in the residency match [28, 29].

We also note that the association of residency cap changes with program growth appears nonlinear, cap increases being associated with program growth and cap decreases under Section 5506 having no statistically significant impact on growth. This pattern is likely reflective of the targeting of cap decreases to those operating below their caps, generating decreases in hospitals’ potential reimbursements but not their realized ones.

While these results suggest that there may be a role for GME funding to play in addressing the primary care and rural physician shortages, there remain elements of this policy approach that require further research. One outstanding question is the extent to which increasing the number of physicians that complete residency training in rural areas results in an increase in the number of attending physicians practicing in those areas. While 54 percent of attending physicians practice in the same state where they completed their residency training, the extent of within-state migration during the transition from medical trainee to attending physician is not clear and warrants further study [30]. Another element that requires research is the extent to which physicians who complete primary care residencies practice primary care as attendings versus pursuing additional GME and subspecializing.^12^

One limitation of our study is that, while we are able to describe the characteristics of hospitals that receive cap increase under Section 5506 because of the closure of a nearby hospital, we are unable to describe the closed hospitals themselves and how they compare to the hospitals that receive their preserved residency slots. Second, the measure of primary care resident utilization from the HCRIS that we use as an outcome variable in our regression analyses does not perfectly correspond to the definition of primary care used in Section 5503, as the former included OB/GYN residents along with primary care ones in its calculation while the latter included general surgery residents. This type of mismeasurement in the specialty-specific resident utilization variables is likely to bias downward the correlation between Section 5503 cap increases and primary care resident counts as it will not capture increases in general surgery resident utilization driven by the provision, and bias upward the estimated correlation of Section 5503 cap increases and non-primary resident counts. Third, our focus is on the period preceding the implementation of many other major ACA policies, including states’ adoption of the Medicaid expansion, which prevents us from speaking about the long-term effects of the provisions studied here. Fourth, our OLS estimates of the relationship between residency cap changes and program size may be biased by unobserved confounders. These estimates should therefore be interpreted as associations rather than casual effects.

Also of importance to consider are the many other policy approaches that have been considered and pursued to try to address the physician shortage issue. These include but are not limited to loan forgiveness programs, scope of practice laws, and adoption of telehealth [31–33]. In 2023, the Consolidated Appropriations Act (CAA) will raise the residency caps of selected teaching hospitals by up to 200 cumulative slots in 2023 using eligibility criteria identical to those used in Section 5503 with the exception of also including as eligible hospitals in states with new medical schools or branch campuses [19]. For this reason, it is perhaps most useful to consider how these alternative policy approaches can be best designed to complement effects we can expect to see from the CAA’s GME provisions given the results presented here on previous GME funding changes. For example, an increase in rural medicine pathways at medical schools, particularly those in areas prioritized by the CAA’s GME provisions, may facilitate improved matching between teaching hospitals treated under these new GME provisions and medical school graduates with a passion for rural medicine.

## Supporting information

S1 Table Detailed summary of teaching hospital characteristics and changes made to Medicare graduate medical education funding as part of ACA Sections 5506 and 5503, 2007. (DOCX)

S2 Table Detailed estimation results for regressions of change in residency program size between 2007 and 2013 on cumulative change in residency funding caps. (DOCX)

S3 Table Detailed estimation results for regressions of change in residency program size between 2007 and 2013 on Section 5506 change in residency funding caps. (DOCX)

S4 Table Detailed estimation results for regressions of change in residency program size between 2007 and 2013 on Section 5503 increases in residency funding caps. (DOCX)

S5 Table Detailed estimation results for regressions of change in residency program size between 2007 and 2013 on Section 5503 decreases in residency funding caps. (DOCX)

## References

[1] The complexities of physician supply and demand: Projections from 2019 to 2034 [Internet]. IHS Markit Ltd; 2021. Available from: https://digirepo.nlm.nih.gov/master/borndig/9918417887306676/9918417887306676.pdf

[2] Bond AM, Casalino LP, Tai-Seale M, Unruh MA, Zhang M, Qian Y, et al. Physician turnover in the United States. Annals of Internal Medicine. 2023;176(7):896–903. doi: 10.7326/M22-2504 37429029

[3] Goroll AH. The future of the US physician workforce—challenges and opportunities. JAMA Network Open. 2021;4(11). doi: 10.1001/jamanetworkopen.2021.34464 34783830

[4] Blumenthal D, Collins SR, Fowler EJ. The Affordable Care Act at 10 years—its coverage and access provisions. New England Journal of Medicine. 2020;382(10):963–9. doi: 10.1056/NEJMhpr1916091 32101659

[5] Frogner BK, Dill JS. Tracking turnover among health care workers during the COVID-19 pandemic. JAMA Health Forum. 2022;3(4). doi: 10.1001/jamahealthforum.2022.0371 35977315 PMC8994131

[6] Health Workforce Shortage Areas [Internet]. Health Resources & Services Administration; 2023. Available from: https://data.hrsa.gov/topics/health-workforce/shortage-areas

[7] Physician Workforce: Caps on Medicare-Funded Graduate Medical Education at Teaching Hospitals [Internet]. Government Accountability Office; 2021. Available from: https://www.gao.gov/products/gao-21-391

[8] Basu S, Berkowitz SA, Phillips RL, Bitton A, Landon BE, Phillips RS. Association of Primary Care physician supply with population mortality in the United States, 2005–2015. JAMA Internal Medicine. 2019;179(4):506. doi: 10.1001/jamainternmed.2018.7624 30776056 PMC6450307

[9] Chang C-H. Primary Care Physician Workforce and Medicare beneficiaries’ health outcomes. JAMA. 2011;305(20):2096. doi: 10.1001/jama.2011.665 21610242 PMC3108147

[10] Plascak JJ, Fisher JL, Paskett ED. Primary care physician supply, insurance type, and late-stage cancer diagnosis. American journal of preventive medicine, 2015;48(2), 174–178. doi: 10.1016/j.amepre.2014.08.014 25441233 PMC4302041

[11] Campbell RJ, Ramirez AM, Perez K, Roetzheim RG. Cervical Cancer rates and the supply of primary care physicians in Florida. Family Medicine. 2003;35(1):60–64. . 12564867

[12] Henry TA. The Physician Shortage Crisis is Here–And So Are Bipartisan Fixes [Internet]. The American Medical Association; 2023. Available from: https://www.ama-assn.org/practice-management/sustainability/physician-shortage-crisis-here-and-so-are-bipartisan-fixes#:~:text=The%20Conrad%20State%2030%20and,help%20ease%20the%20physician%20shortage

[13] NHS Long Term Workforce Plan [Internet]. National Health Service; 2024. Available from: https://www.england.nhs.uk/long-read/nhs-long-term-workforce-plan-2/

[14] Chandrasiri S. How to Solve Australia’s Health Workforce Shortage [Internet]. InSight; 2023. Available from: https://insightplus.mja.com.au/2023/18/how-to-solve-australias-health-workforce-shortage/

[15] Locations and Types of Graduate Training Were Largely Unchanged, and Federal Efforts May Not be Sufficient to Meet Needs [Internet]. United States Government Accountability Office; 2017. Available from: https://www.gao.gov/assets/gao-17-411.pdf

[16] Programs and Residents Increased during Transition to Single Accreditor; Distribution Largely Unchanged [Internet]. United States Government Accountability Office; 2021. Available from: https://www.gao.gov/assets/gao-21-329.pdf

[17] Medicare Graduate Medical Education Payment: An Overview [Internet]. Congressional Research Service; 2022. Available from: https://crsreports.congress.gov/product/pdf/IF/IF10960

[18] Federal Support for Graduate Medical Education: An Overview [Internet]. Congressional Research Service; 2018. Available from: https://crsreports.congress.gov/product/pdf/R/R44376

[19] Direct Graduate Medical Education (DGME) [Internet]. CMS; 2023. Available from: https://www.cms.gov/Medicare/Medicare-Fee-for-Service-Payment/AcuteInpatientPPS/DGME

[20] Rural Hospital Closures Threaten Access [Internet]. American Hospital Association; 2022. Available from: https://www.aha.org/system/files/media/file/2022/09/rural-hospital-closures-threaten-access-report.pdf

[21] Hayes OW, Scaglione J, Hutchinson CP, Zhorzholiani I. Graduate Medical Education Enhancement and the Consolidated Appropriations Act, 2021. J Grad Med Educ. 2021 Oct;13(5):650–653. doi: 10.4300/JGME-D-21-00467.1 34721793 PMC8527944

[22] Chandra A, Khullar D, Wilensky GR. The Economics of Graduate Medical Education. New England Journal of Medicine. 2014;370(25):2357–60. doi: 10.1056/NEJMp1402468 24826947

[23] Grover A, Slavin PL, Willson P. The Economics of Academic Medical Centers. New England Journal of Medicine. 2014;370(25):2360–2. doi: 10.1056/NEJMp1403609 24826948

[24] Cost Reports by Fiscal Year: 2007. [Internet]. CMS; 2007. Available from: https://www.cms.gov/research-statistics-data-and-systems/downloadable-public-use-files/cost-reports/cost-reports-by-fiscal-year-items/hosp-dl-2007

[25] Cost Reports by Fiscal Year: 2013. [Internet]. CMS; 2013. Available from: https://www.cms.gov/research-statistics-data-and-systems/downloadable-public-use-files/cost-reports/cost-reports-by-fiscal-year-items/hospital10-dl-2013

[26] § 412.105 Special treatment: Hospitals that incur indirect costs for graduate medical education programs. [Internet]. Code of Federal Regulations; 1985. Available from: https://www.ecfr.gov/current/title-42/chapter-IV/subchapter-B/part-412/subpart-G/section-412.105

[27] Medicare Program: Hospital Outpatient Prospective Payment System and CY 2011 Payment Rates, 75 Fed. Reg. 72177 (November 24, 2010) (to be codified at 42 CFR Parts 410, 411, 412, et al.).

[28] Melcher ML, Ashlagi I, Wapnir I. Matching for fellowship interviews. JAMA. 2018;320(16):1639. doi: 10.1001/jama.2018.13080 30422279

[29] Gadepalli SK, Downard CD, Thatch KA, Islam S, Azarow KS, Chen MK, et al. Association of Pediatric Surgery Training Program Directors. The effort and outcomes of the Pediatric Surgery match process: Are we interviewing too many? J Pediatr Surg. 2015 Nov;50(11):1954–7. doi: 10.1016/j.jpedsurg.2015.06.008 26165158

[30] Table C6: Physician Retention in State of Residency Training, by State [Internet]. AAMC; 2023. Available from: https://www.aamc.org/data-reports/students-residents/data/table-c6-physician-retention-state-residency-training-state

[31] Pathman DE, Konrad TR, King TS, Taylor DH, Koch GG. Outcomes of states’ scholarship, loan repayment, and related programs for physicians. Medical Care. 2004;42(6):560–8. doi: 10.1097/01.mlr.0000128003.81622.ef 15167324

[32] Dower C, Moore J, Langelier M. It is time to restructure health professions scope-of-practice regulations to remove barriers to care. Health Affairs. 2013;32(11):1971–6. doi: 10.1377/hlthaff.2013.0537 24191088

[33] Severin C, Curry M. Telehealth Funding: Transforming Primary Care and Achieving Digital Health Equity for Underresourced Populations [Internet]. Health Affairs; 2021. Available from: https://www.healthaffairs.org/content/forefront/telehealth-funding-transforming-primary-care-and-achieving-digital-health-equity

