# Supplementary material for: Impact of federal funding for graduate medical education on residency program size: Evidence from the Affordable Care Act
Source: PLoS One. 2025 Feb 10;20(2):e0318626. doi: 10.1371/journal.pone.0318626 (PMC11809784; doi:10.1371/journal.pone.0318626)
Supplement: S1 Table — (DOCX) [file pone.0318626.s001.docx]

Impact of federal funding for graduate medical education on residency program size: Evidence from the Affordable Care Act (Supplement)

By Cici McNamara and Tehreem Hussain

Our interest is in how changes to teaching hospitals’ resident caps are associated with residency program growth. We estimate these associations with an OLS regression of the form

$${\Delta NumRes}_{h}= \alpha+ \beta\Delta{ResCap}_{h}+ \mu_{s(h)}+ \varepsilon_{h}.$$

Here, ${\Delta NumRes}_{h}$ is the change in hospital *h*’s number of full-time equivalent (FTE) residents between the years 2013 and 2007: ${\Delta NumRes}_{h}= {NumRes}_{h,t=2013}- {NumRes}_{h,t=2007}$. $\Delta{ResCap}_{h}$ is the change in hospital *h*’s residency funding cap implemented under Sections 5506 or 5503. $\mu_{s(h)}$ are fixed effects for the state *s* in which hospital *h* is located. Two teaching hospitals that are the only teaching hospitals in their respective states are grouped together under a single fixed effect.

We estimate the regression model above for four specifications defined by the sample-resident cap measure pair used. The first specification uses the full balanced panel of teaching hospitals (i.e., all hospitals active in both 2007 and 2013) and defines $\Delta{ResCap}_{h}$ as the cumulative change in hospital *h*’s residency cap under ACA Sections 5503 and 5506. The second specification includes only hospitals that received Section 5506 resident cap increases and those that did not receive a resident cap change under any ACA provision and defines $\Delta{ResCap}_{h}$ as the residency cap change stemming from Section 5506 alone. The third specification includes only hospitals that received Section 5503 resident cap increases and those that did not receive a resident cap change under any ACA provision and defines $\Delta{ResCap}_{h}$ as the residency cap increase stemming from Section 5503 alone. The fourth specification includes only hospitals that received Section 5503 resident cap decreases and those that did not receive a resident cap change under any ACA provision and defines $\Delta{ResCap}_{h}$ as the residency cap decrease stemming from Section 5503 alone. We estimate each of these four regression specifications using four measures of FTE resident counts: direct graduate medical education (DGME) FTEs, indirect graduate medical education (IME) FTEs, primary care DGME FTEs, and non-primary care DGME FTEs. All regressions are weighted by 2007 hospital discharge volumes. California, the largest state in the sample in terms of number of teaching hospitals, is the reference group for state fixed effects. Fixed effect estimates will be missing for states with no teaching hospitals in the subsamples.

Supplement table 1 provides detailed summary statistics of variables of interest for the full sample of teaching hospitals active in 2007 (the sample that is used in Exhibit 2 in the main text). Supplement tables 2-5 presents the full estimation results for regression specifications 1-4, respectively.

**S1 Table: Detailed summary of teaching hospital characteristics and changes made to Medicare Graduate Medical Education funding as part of ACA Sections 5506 and 5503, 2007**

|  | Mean | S.d. | p10 | p50 | p90 |
| --- | --- | --- | --- | --- | --- |
| General practice physicians per thousand | 3.3 | 1.7 | 1.8 | 3.0 | 5.7 |
| Annual discharges (thous.) | 15.4 | 12.2 | 2.4 | 13.1 | 30.3 |
| Size of resident cap change |  |  |  |  |  |
| DGME | 0.5 | 6.1 | -1.0 | 0.0 | 0.0 |
| IME | 0.5 | 5.7 | -0.3 | 0.0 | 0.0 |
| Baseline resident cap |  |  |  |  |  |
| DGME | 68.2 | 118.9 | 0.0 | 20.1 | 213.6 |
| IME | 61.4 | 113.2 | 0.0 | 15.9 | 201.6 |
| Baseline number of residents trained |  |  |  |  |  |
| DGME | 73.2 | 130.2 | 0.0 | 19.6 | 229.0 |
| IME | 66.3 | 124.1 | 0.0 | 16.9 | 211.7 |
| Baseline GME payment |  |  |  |  |  |
| DGME | 2.3 | 4.2 | 0.0 | 0.7 | 6.7 |
| IME | 4.6 | 9.1 | 0.0 | 1.1 | 13.7 |
| Observations | 1,288 | | | | |

Notes: Data on baseline characteristics corresponds to 2007. Cells contain means. Sample is all teaching hospitals active in 2007. The resident cap change measure is the cumulative change across Sections 5506 and 5503. General practice physicians per thousand is computed at the county-level. Statistics for area-level physician supply do not include observations from Puerto Rico.
