# Supplementary material for: Impact of federal funding for graduate medical education on residency program size: Evidence from the Affordable Care Act
Source: PLoS One. 2025 Feb 10;20(2):e0318626. doi: 10.1371/journal.pone.0318626 (PMC11809784; doi:10.1371/journal.pone.0318626)
Supplement: S2 Table — (DOCX) [file pone.0318626.s002.docx]

**S2 Table: Detailed estimation results for regressions of change in residency program size between 2007 and 2013 on cumulative change in residency funding caps**

|  | (1)  DGME residents | | (2)  DGME primary care residents | | (3)  DGME non-primary care residents | | (4)  IME residents | |
| --- | --- | --- | --- | --- | --- | --- | --- | --- |
| Cumulative resident cap change | 0.98 | (0.22) | 0.27 | (0.09) | 0.61 | (0.15) | 0.99 | (0.25) |
| **State/territory fixed effects** |  |  |  |  |  |  |  |  |
| CA (reference) | -- |  | -- |  | -- |  | -- |  |
| AL | -3.62 | (18.72) | -0.92 | (3.59) | -2.91 | (11.95) | -8.16 | (15.92) |
| AR | -10.90 | (3.91) | -0.07 | (1.50) | -9.07 | (2.42) | -11.58 | (3.52) |
| AZ | -2.28 | (10.37) | 0.53 | (4.85) | -3.50 | (5.03) | -3.54 | (10.26) |
| CO | -4.76 | (6.37) | 1.41 | (1.83) | -4.43 | (4.58) | -4.77 | (6.77) |
| CT | -15.61 | (3.80) | -3.05 | (1.96) | -6.77 | (2.64) | -13.25 | (3.28) |
| DC | -11.54 | (11.74) | -0.26 | (5.01) | -10.40 | (10.37) | -15.60 | (12.33) |
| DE | 4.92 | (7.61) | 2.51 | (2.09) | 5.85 | (6.19) | 5.79 | (7.08) |
| FL | -2.85 | (5.60) | 1.92 | (2.62) | 0.82 | (4.45) | -4.15 | (6.30) |
| GA | -0.69 | (5.15) | 0.88 | (3.60) | 0.07 | (3.79) | -0.55 | (5.07) |
| HI | -11.07 | (6.37) | -0.14 | (2.78) | -7.76 | (3.73) | -10.13 | (6.56) |
| IA | -3.19 | (7.89) | -2.79 | (2.76) | -0.37 | (8.10) | -1.12 | (8.39) |
| ID | -13.48 | (5.55) | 3.52 | (2.44) | -11.51 | (2.37) | -12.44 | (5.55) |
| IL | -5.15 | (4.64) | 0.92 | (1.84) | -3.68 | (3.59) | -4.08 | (4.93) |
| IN | -15.42 | (4.49) | -3.86 | (3.23) | -7.00 | (6.43) | -9.00 | (7.45) |
| KS | -6.35 | (7.67) | -2.07 | (1.90) | -4.69 | (6.58) | -6.25 | (6.71) |
| KY | -4.05 | (10.49) | 0.04 | (3.00) | -3.27 | (6.62) | -3.77 | (8.82) |
| LA | -11.69 | (18.88) | 3.57 | (8.17) | -12.62 | (9.63) | 8.54 | (11.52) |
| MA | -3.23 | (5.84) | -0.66 | (3.40) | -1.40 | (4.50) | 2.07 | (5.79) |
| MD | 19.18 | (16.23) | 0.07 | (4.22) | 6.63 | (9.17) | 16.78 | (16.75) |
| ME | -8.24 | (6.82) | 1.96 | (3.77) | -6.98 | (3.69) | -11.91 | (4.19) |
| MI | 7.09 | (6.05) | 2.75 | (2.77) | 5.35 | (4.34) | 6.50 | (6.37) |
| MN | -10.66 | (5.87) | -0.39 | (1.60) | -7.86 | (3.64) | -10.00 | (5.55) |
| MO | 0.93 | (7.08) | 2.33 | (1.95) | 1.20 | (6.54) | -0.10 | (6.58) |
| MS | 5.04 | (16.87) | 5.09 | (5.81) | 2.28 | (10.58) | 6.15 | (16.76) |
| MT | -22.96 | (7.91) | -2.69 | (2.90) | -16.88 | (4.59) | -22.11 | (8.41) |
| NC | 9.86 | (8.76) | 0.14 | (4.36) | 8.53 | (8.66) | 9.97 | (9.64) |
| ND | -11.75 | (4.13) | -0.51 | (1.61) | -6.88 | (3.04) | -8.61 | (3.75) |
| NE | -8.28 | (5.14) | -0.66 | (1.50) | -4.95 | (4.87) | -7.26 | (5.77) |
| NH | 2.70 | (11.99) | 3.69 | (4.00) | -4.40 | (4.68) | 5.11 | (12.11) |
| NJ | -8.79 | (4.62) | 0.65 | (2.49) | -6.95 | (3.32) | -7.09 | (4.64) |
| NM | -4.89 | (9.19) | 2.30 | (3.97) | -5.31 | (5.07) | -2.50 | (10.18) |
| NV | -3.74 | (18.73) | 10.44 | (9.48) | -12.54 | (7.97) | -5.19 | (17.70) |
| NY | 12.55 | (8.81) | 5.76 | (4.65) | 6.64 | (4.84) | 16.97 | (10.19) |
| OH | 8.26 | (8.63) | 6.79 | (3.23) | -1.43 | (4.72) | 10.23 | (9.99) |
| OK | -8.46 | (7.97) | -0.60 | (3.26) | -6.59 | (4.61) | -4.55 | (7.32) |
| OR | 2.59 | (14.21) | 2.46 | (2.23) | -0.70 | (9.80) | 5.90 | (15.91) |
| PA | 2.08 | (6.31) | -1.02 | (2.35) | 2.35 | (4.70) | 0.22 | (4.79) |
| PR | -15.63 | (3.99) | -3.22 | (2.46) | -10.53 | (3.47) | -16.95 | (3.50) |
| RI | -15.88 | (3.70) | 0.08 | (3.29) | -13.09 | (4.67) | -11.61 | (3.20) |
| SC | -0.51 | (6.71) | 1.29 | (2.46) | -1.30 | (5.18) | -0.17 | (6.04) |
| SD | -8.43 | (4.84) | 8.15 | (5.54) | -13.21 | (4.49) | -2.06 | (5.76) |
| TN | 5.86 | (11.57) | -2.50 | (2.68) | 7.61 | (10.09) | 10.29 | (13.69) |
| TX | 5.71 | (6.17) | 2.56 | (2.00) | 3.31 | (4.61) | 0.05 | (5.61) |
| UT | 0.28 | (11.94) | -1.65 | (2.23) | 2.90 | (12.26) | -2.44 | (10.85) |
| VA | -4.44 | (5.63) | 0.39 | (3.91) | -4.80 | (3.15) | -1.02 | (6.50) |
| WA | -3.70 | (5.74) | 0.50 | (2.30) | -2.42 | (5.07) | -5.40 | (5.40) |
| WI | 2.68 | (7.67) | 4.94 | (4.14) | -1.98 | (4.90) | 0.34 | (6.87) |
| WV | -3.92 | (4.82) | -0.11 | (2.51) | -0.16 | (4.39) | -3.38 | (4.87) |
| WY | -15.64 | (3.09) | -2.62 | (1.09) | -10.64 | (2.07) | -14.51 | (3.10) |
| States with < 2 teaching hospitals | 0.05 | (9.45) | 0.05 | (1.11) | -1.72 | (6.31) | -0.14 | (8.70) |
| Constant | 15.64 | (3.09) | 2.62 | (1.09) | 10.64 | (2.07) | 14.57 | (3.07) |
| Number of observations | 1,216 | | | | | | | |

Notes: All regressions were performed on the sample of all teaching hospitals in the balanced panel.
