# Supplementary material for: Impact of federal funding for graduate medical education on residency program size: Evidence from the Affordable Care Act
Source: PLoS One. 2025 Feb 10;20(2):e0318626. doi: 10.1371/journal.pone.0318626 (PMC11809784; doi:10.1371/journal.pone.0318626)
Supplement: S3 Table — (DOCX) [file pone.0318626.s003.docx]

**S3 Table: Detailed estimation results for regressions of change in residency program size between 2007 and 2013 on Section 5506 change in residency funding caps**

|  | (1)  DGME residents | | (2)  DGME primary care residents | | (3)  DGME non-primary care residents | | (4)  IME residents | |
| --- | --- | --- | --- | --- | --- | --- | --- | --- |
| Section 5506 resident cap change | 1.31 | (0.43) | 0.27 | (0.15) | 0.86 | (0.26) | 1.31 | (0.47) |
| **State/territory fixed effects** |  |  |  |  |  |  |  |  |
| CA (reference) | -- |  | -- |  | -- |  | -- |  |
| AL | 17.85 | (31.70) | 2.35 | (5.72) | 11.33 | (19.61) | 10.19 | (25.54) |
| AR | -11.63 | (4.12) | -0.18 | (1.61) | -9.49 | (2.63) | -11.90 | (3.68) |
| AZ | -0.56 | (12.87) | 1.34 | (5.97) | -2.92 | (6.27) | -1.94 | (12.74) |
| CO | 1.12 | (9.23) | 3.62 | (2.42) | -1.00 | (6.93) | 0.82 | (10.04) |
| CT | -14.42 | (3.44) | -3.46 | (2.16) | -7.22 | (2.94) | -13.91 | (3.47) |
| DC | -6.91 | (4.90) | 6.80 | (3.78) | -5.19 | (4.00) | -2.60 | (5.32) |
| DE | 6.48 | (6.95) | 2.96 | (2.01) | 7.33 | (5.63) | 7.73 | (6.32) |
| FL | -4.97 | (6.06) | -0.37 | (3.70) | 1.20 | (7.32) | -6.87 | (4.68) |
| GA | -3.17 | (5.77) | -2.02 | (3.73) | -0.35 | (4.55) | -3.03 | (5.57) |
| HI | -11.80 | (6.53) | -0.25 | (2.85) | -8.18 | (3.88) | -10.45 | (6.67) |
| IA | -1.23 | (9.45) | -3.35 | (3.40) | 1.70 | (9.90) | 1.27 | (10.10) |
| ID |  |  |  |  |  |  |  |  |
| IL | -6.62 | (5.58) | -0.22 | (2.11) | -3.36 | (4.18) | -5.09 | (5.90) |
| IN | -16.11 | (3.49) | -0.88 | (1.59) | -11.59 | (2.52) | -13.93 | (3.53) |
| KS | -4.82 | (9.78) | -2.21 | (2.40) | -3.63 | (8.56) | -4.63 | (8.45) |
| KY | -4.40 | (11.26) | -0.23 | (3.24) | -3.24 | (7.12) | -3.76 | (9.44) |
| LA | -13.56 | (30.73) | 4.02 | (13.18) | -14.55 | (15.58) | 20.40 | (16.04) |
| MA | -1.42 | (6.65) | 0.01 | (4.03) | -0.19 | (5.26) | 4.93 | (6.40) |
| MD | 18.45 | (16.37) | -0.04 | (4.29) | 6.21 | (9.28) | 16.46 | (16.88) |
| ME | -6.52 | (8.30) | 2.53 | (5.02) | -5.31 | (4.02) | -11.80 | (4.84) |
| MI | 6.46 | (6.64) | 2.69 | (3.07) | 5.14 | (4.81) | 6.35 | (7.01) |
| MN | -17.76 | (4.59) | -2.00 | (1.58) | -12.26 | (2.93) | -16.36 | (4.77) |
| MO | 1.35 | (7.63) | 2.58 | (2.15) | 1.62 | (7.11) | 0.46 | (7.07) |
| MS | -18.53 | (3.46) | -4.67 | (1.31) | -11.01 | (2.58) | -17.05 | (3.37) |
| MT |  |  |  |  |  |  |  |  |
| NC | 12.72 | (9.42) | 0.50 | (5.02) | 11.18 | (9.64) | 13.51 | (10.49) |
| ND | -12.47 | (4.33) | -0.62 | (1.71) | -7.30 | (3.22) | -8.92 | (3.90) |
| NE | -8.39 | (6.24) | -1.19 | (1.70) | -4.33 | (6.09) | -6.14 | (6.95) |
| NH | 1.97 | (12.13) | 3.58 | (4.07) | -4.82 | (4.82) | 4.79 | (12.23) |
| NJ | -9.15 | (5.68) | 0.69 | (3.05) | -7.33 | (3.93) | -7.36 | (5.76) |
| NM | -16.96 | (3.49) | -3.25 | (1.38) | -11.20 | (2.39) | -15.36 | (3.36) |
| NV | 16.14 | (31.15) | 14.01 | (18.27) | -0.66 | (8.72) | 14.51 | (28.64) |
| NY | 13.62 | (10.64) | 7.32 | (5.81) | 6.32 | (5.77) | 19.67 | (12.32) |
| OH | 6.34 | (9.16) | 7.03 | (3.50) | -3.18 | (4.89) | 7.98 | (10.49) |
| OK | -12.69 | (11.03) | -2.64 | (4.36) | -9.05 | (6.35) | -6.51 | (10.01) |
| OR | 3.61 | (15.47) | 2.81 | (2.39) | -0.15 | (10.74) | 7.54 | (17.31) |
| PA | 3.02 | (7.22) | -1.38 | (2.74) | 3.63 | (5.40) | 0.69 | (5.40) |
| PR | -19.53 | (4.57) | 1.48 | (6.72) | -8.31 | (2.66) | -22.37 | (3.85) |
| RI | -16.56 | (4.00) | -0.09 | (3.60) | -13.39 | (5.11) | -11.85 | (3.38) |
| SC | -1.70 | (6.74) | 0.92 | (2.66) | -1.82 | (5.08) | -1.02 | (6.06) |
| SD | -17.99 | (3.35) | -2.47 | (1.24) | -11.07 | (2.32) | -14.88 | (3.25) |
| TN | 7.04 | (14.09) | -3.91 | (3.08) | 10.08 | (12.15) | 12.67 | (16.67) |
| TX | 9.40 | (7.58) | 2.59 | (2.46) | 7.01 | (5.81) | 1.39 | (6.92) |
| UT | 0.25 | (12.48) | -1.66 | (2.39) | 3.03 | (12.83) | -2.22 | (11.32) |
| VA | -3.11 | (6.49) | 0.68 | (4.72) | -4.10 | (3.58) | 1.21 | (7.46) |
| WA | -2.32 | (7.13) | 0.13 | (2.89) | -0.64 | (6.41) | -4.25 | (6.62) |
| WI | 2.62 | (9.04) | 3.71 | (4.71) | -1.02 | (5.80) | -0.54 | (7.97) |
| WV | -3.87 | (5.07) | -0.06 | (2.73) | 0.14 | (4.62) | -2.96 | (5.07) |
| WY | -16.36 | (3.35) | -2.73 | (1.24) | -11.07 | (2.32) | -14.83 | (3.27) |
| States with < 2 teaching hospitals | -0.68 | (9.59) | -0.06 | (1.26) | -2.14 | (6.43) | -0.45 | (8.81) |
| Constant | 16.36 | (3.35) | 2.73 | (1.24) | 11.07 | (2.32) | 14.88 | (3.25) |
| Number of observations | 913 | | | | | | | |

Notes: All regressions were performed on the subsample of teaching hospitals that participated in Section 5506 and those that did receive any type of cap change. There are no teaching hospitals from Idaho in this subsample, so no state fixed effect for Idaho is estimated.
