# Supplementary material for: Impact of federal funding for graduate medical education on residency program size: Evidence from the Affordable Care Act
Source: PLoS One. 2025 Feb 10;20(2):e0318626. doi: 10.1371/journal.pone.0318626 (PMC11809784; doi:10.1371/journal.pone.0318626)
Supplement: S4 Table — (DOCX) [file pone.0318626.s004.docx]

**S4 Table: Detailed estimation results for regressions of change in residency program size between 2007 and 2013 on Section 5503 increases in residency funding caps**

|  | (1)  DGME residents | | (2)  DGME primary care residents | | (3)  DGME non-primary care residents | | (4)  IME residents | |
| --- | --- | --- | --- | --- | --- | --- | --- | --- |
| Section 5503 resident cap increase | 0.75 | (0.21) | 0.33 | (0.10) | 0.37 | (0.17) | 0.79 | (0.23) |
| **State/territory fixed effects** |  |  |  |  |  |  |  |  |
| CA (reference) | -- |  | -- |  | -- |  | -- |  |
| AL | -21.35 | (6.82) | -3.73 | (3.43) | -13.38 | (4.06) | -22.26 | (7.46) |
| AR | -11.63 | (4.12) | -0.18 | (1.61) | -9.49 | (2.64) | -11.90 | (3.68) |
| AZ | 7.09 | (13.80) | 6.72 | (5.78) | 0.40 | (6.73) | 6.13 | (13.50) |
| CO | 1.12 | (9.24) | 3.62 | (2.43) | -1.00 | (6.94) | 0.82 | (10.04) |
| CT | -14.66 | (3.45) | -3.58 | (2.27) | -7.30 | (3.02) | -14.22 | (3.48) |
| DC | -8.72 | (9.92) | -1.36 | (5.40) | -7.06 | (8.55) | -12.73 | (10.57) |
| DE | 6.48 | (6.95) | 2.96 | (2.02) | 7.33 | (5.64) | 7.73 | (6.33) |
| FL | -1.85 | (6.38) | 1.87 | (3.04) | 2.29 | (5.18) | -3.23 | (7.16) |
| GA | -4.28 | (5.79) | -1.70 | (3.85) | -1.35 | (4.57) | -2.52 | (5.69) |
| HI | -11.80 | (6.53) | -0.25 | (2.86) | -8.18 | (3.89) | -10.45 | (6.68) |
| IA | -1.23 | (9.46) | -3.35 | (3.40) | 1.70 | (9.91) | 1.27 | (10.11) |
| ID | -18.18 | (3.44) | 0.69 | (1.70) | -12.27 | (2.39) | -16.83 | (3.35) |
| IL | -1.82 | (5.72) | 0.99 | (2.13) | -0.40 | (4.42) | 0.39 | (6.18) |
| IN | -14.60 | (5.01) | -4.88 | (3.48) | -5.27 | (7.16) | -7.35 | (8.08) |
| KS | -4.82 | (9.79) | -2.21 | (2.40) | -3.63 | (8.57) | -4.63 | (8.46) |
| KY | -4.40 | (11.27) | -0.23 | (3.24) | -3.24 | (7.13) | -3.76 | (9.45) |
| LA | -11.90 | (21.73) | 4.07 | (9.38) | -13.19 | (11.09) | 11.42 | (12.86) |
| MA | -1.42 | (6.66) | 0.01 | (4.03) | -0.19 | (5.26) | 4.93 | (6.41) |
| MD | 18.45 | (16.39) | -0.04 | (4.29) | 6.21 | (9.29) | 16.46 | (16.90) |
| ME | -6.52 | (8.31) | 2.53 | (5.03) | -5.31 | (4.03) | -11.80 | (4.84) |
| MI | 6.40 | (6.75) | 2.81 | (3.11) | 4.96 | (4.92) | 6.33 | (7.13) |
| MN | -17.76 | (4.60) | -2.00 | (1.58) | -12.26 | (2.94) | -16.36 | (4.78) |
| MO | 0.72 | (7.90) | 2.08 | (2.15) | 1.26 | (7.38) | -0.80 | (7.27) |
| MS | 6.56 | (18.63) | 4.23 | (5.32) | 4.33 | (12.54) | 7.82 | (18.30) |
| MT | -21.35 | (6.85) | -3.45 | (3.35) | -14.83 | (3.73) | -20.33 | (7.39) |
| NC | 8.01 | (9.05) | -0.07 | (5.48) | 7.59 | (9.89) | 8.76 | (10.38) |
| ND | -12.47 | (4.34) | -0.62 | (1.72) | -7.30 | (3.22) | -8.92 | (3.91) |
| NE | -8.39 | (6.25) | -1.19 | (1.70) | -4.33 | (6.09) | -6.14 | (6.96) |
| NH | 1.97 | (12.14) | 3.58 | (4.07) | -4.82 | (4.82) | 4.79 | (12.24) |
| NJ | -10.81 | (5.89) | 2.33 | (4.51) | -10.76 | (2.66) | -8.30 | (5.90) |
| NM | -4.17 | (10.37) | 1.79 | (3.70) | -4.20 | (6.34) | -1.51 | (11.21) |
| NV | -0.01 | (18.67) | 9.09 | (10.22) | -8.25 | (7.13) | -1.50 | (17.61) |
| NY | 0.76 | (5.97) | 0.39 | (3.30) | 1.70 | (4.06) | 4.13 | (6.50) |
| OH | 6.34 | (9.16) | 7.03 | (3.50) | -3.18 | (4.90) | 7.98 | (10.50) |
| OK | -12.87 | (10.52) | -2.58 | (4.16) | -9.21 | (6.08) | -6.95 | (9.53) |
| OR | 3.61 | (15.48) | 2.81 | (2.39) | -0.15 | (10.75) | 7.54 | (17.33) |
| PA | 1.06 | (7.94) | -1.54 | (3.10) | 2.68 | (5.88) | -1.82 | (5.59) |
| PR | -17.44 | (4.44) | -4.56 | (4.56) | -6.02 | (4.19) | -17.29 | (4.92) |
| RI | -16.56 | (4.00) | -0.09 | (3.60) | -13.39 | (5.11) | -11.85 | (3.38) |
| SC | -6.56 | (5.56) | 1.60 | (2.82) | -6.40 | (3.00) | -5.08 | (5.17) |
| SD | -6.68 | (5.88) | 7.35 | (5.24) | -11.01 | (3.81) | -0.15 | (6.08) |
| TN | 7.04 | (14.10) | -3.91 | (3.08) | 10.08 | (12.16) | 12.67 | (16.68) |
| TX | 9.40 | (7.59) | 2.59 | (2.46) | 7.01 | (5.81) | 1.39 | (6.93) |
| UT | 0.25 | (12.49) | -1.66 | (2.40) | 3.03 | (12.84) | -2.22 | (11.33) |
| VA | -3.30 | (6.43) | 0.68 | (4.66) | -4.23 | (3.56) | 0.98 | (7.38) |
| WA | -2.32 | (7.13) | 0.13 | (2.89) | -0.64 | (6.42) | -4.25 | (6.63) |
| WI | 3.90 | (8.63) | 5.47 | (4.70) | -1.36 | (5.56) | 1.44 | (7.74) |
| WV | -3.87 | (5.08) | -0.06 | (2.74) | 0.14 | (4.62) | -2.96 | (5.08) |
| WY | -16.36 | (3.35) | -2.73 | (1.24) | -11.07 | (2.32) | -14.83 | (3.28) |
| States with < 2 teaching hospitals | -0.68 | (9.60) | -0.06 | (1.26) | -2.14 | (6.44) | -0.45 | (8.82) |
| Constant | 16.36 | (3.35) | 2.73 | (1.24) | 11.07 | (2.32) | 14.88 | (3.25) |
| Number of observations | 921 | | | | | | | |

Notes: All regressions were performed on the subsample of teaching hospitals that received a cap increase under Section 5503 and those that did receive any type of cap change.
