# Supplementary material for: Impact of federal funding for graduate medical education on residency program size: Evidence from the Affordable Care Act
Source: PLoS One. 2025 Feb 10;20(2):e0318626. doi: 10.1371/journal.pone.0318626 (PMC11809784; doi:10.1371/journal.pone.0318626)
Supplement: S5 Table — (DOCX) [file pone.0318626.s005.docx]

**S5 Table: Detailed estimation results for regressions of change in residency program size between 2007 and 2013 on Section 5503 decreases in residency funding caps**

|  | (1)  DGME residents | | (2)  DGME primary care residents | | (3)  DGME non-primary care residents | | (4)  IME residents | |
| --- | --- | --- | --- | --- | --- | --- | --- | --- |
| Section 5503 resident cap decrease | 0.19 | (0.33) | -0.08 | (0.07) | 0.25 | (0.23) | 0.12 | (0.36) |
| **State/territory fixed effects** |  |  |  |  |  |  |  |  |
| CA (reference) | -- |  | -- |  | -- |  | -- |  |
| AL | -14.45 | (4.38) | -3.12 | (2.15) | -7.77 | (2.27) | AL | -13.46 |
| AR | -9.95 | (3.73) | 0.36 | (1.47) | -8.63 | (2.33) | AR | -10.53 |
| AZ | 3.39 | (11.04) | 4.94 | (4.63) | -1.25 | (5.40) | AZ | 2.69 |
| CO | -4.10 | (6.29) | 1.71 | (1.82) | -4.13 | (4.55) | -4.03 | (6.68) |
| CT | -15.42 | (3.84) | -2.86 | (2.01) | -6.61 | (2.64) | -12.91 | (3.02) |
| DC | -5.24 | (4.57) | 7.33 | (3.70) | -4.33 | (3.80) | -1.23 | (5.04) |
| DE | 5.62 | (7.72) | 2.83 | (2.14) | 6.17 | (6.26) | 6.57 | (7.19) |
| FL | -2.88 | (5.06) | -0.87 | (2.97) | 2.33 | (5.85) | -4.47 | (4.14) |
| GA | -2.12 | (4.95) | 0.94 | (3.64) | -1.00 | (3.73) | -0.78 | (4.90) |
| HI | -10.12 | (6.27) | 0.29 | (2.76) | -7.32 | (3.67) | -9.07 | (6.44) |
| IA | -2.32 | (7.82) | -2.40 | (2.74) | 0.03 | (8.09) | -0.16 | (8.31) |
| ID | 1.20 | (2.88) | 11.24 | (1.03) | -7.84 | (1.97) | 2.34 | (2.81) |
| IL | -2.16 | (4.68) | 2.56 | (1.84) | -2.60 | (3.66) | -0.42 | (5.01) |
| IN | -14.01 | (3.02) | -0.77 | (1.41) | -10.18 | (2.17) | -11.94 | (3.05) |
| KS | -5.79 | (7.62) | -1.82 | (1.88) | -4.43 | (6.56) | -5.64 | (6.63) |
| KY | -3.18 | (10.44) | 0.44 | (2.98) | -2.87 | (6.60) | -2.81 | (8.75) |
| LA | -11.90 | (25.41) | 3.45 | (10.97) | -12.70 | (12.87) | 16.23 | (14.15) |
| MA | -2.45 | (5.74) | -0.31 | (3.40) | -1.04 | (4.46) | 2.94 | (5.67) |
| MD | 20.13 | (16.21) | 0.49 | (4.22) | 7.06 | (9.16) | 17.83 | (16.72) |
| ME | -7.60 | (6.84) | 2.24 | (3.80) | -6.69 | (3.69) | -11.20 | (4.07) |
| MI | 8.97 | (6.21) | 3.33 | (2.92) | 6.49 | (4.50) | 8.42 | (6.57) |
| MN | -10.23 | (5.63) | -0.20 | (1.54) | -7.67 | (3.53) | -9.53 | (5.26) |
| MO | 1.03 | (7.24) | 2.21 | (1.92) | 1.15 | (6.75) | -0.47 | (6.64) |
| MS | -16.85 | (3.00) | -4.14 | (1.12) | -10.15 | (2.27) | -15.68 | (2.93) |
| MT |  |  |  |  |  |  |  |  |
| NC | 6.38 | (8.22) | 0.02 | (4.69) | 5.58 | (8.69) | 6.55 | (9.30) |
| ND | -10.80 | (3.97) | -0.09 | (1.57) | -6.44 | (2.97) | -7.55 | (3.52) |
| NE | -7.50 | (5.04) | -0.31 | (1.46) | -4.58 | (4.85) | -6.39 | (5.67) |
| NH | 3.65 | (11.95) | 4.12 | (4.00) | -3.96 | (4.64) | 6.16 | (12.06) |
| NJ | -10.21 | (4.53) | 2.36 | (3.25) | -9.78 | (2.22) | -7.65 | (4.44) |
| NM | -15.29 | (3.03) | -2.71 | (1.20) | -10.34 | (2.06) | -13.98 | (2.92) |
| NV | 17.81 | (30.96) | 14.54 | (18.17) | 0.20 | (8.60) | 15.88 | (28.47) |
| NY | 0.79 | (4.81) | 0.27 | (2.39) | 1.76 | (3.32) | 2.92 | (5.12) |
| OH | 9.12 | (8.56) | 7.17 | (3.22) | -1.04 | (4.68) | 11.18 | (9.92) |
| OK | -7.59 | (8.11) | -0.33 | (3.32) | -6.10 | (4.69) | -3.50 | (7.39) |
| OR | 3.54 | (14.18) | 2.89 | (2.21) | -0.26 | (9.80) | 6.95 | (15.89) |
| PA | 0.93 | (6.82) | -0.80 | (2.62) | 1.74 | (5.07) | -1.23 | (4.78) |
| PR | -16.07 | (4.65) | -2.25 | (3.17) | -12.46 | (3.88) | -18.92 | (3.88) |
| RI | -15.07 | (3.51) | 0.45 | (3.29) | -12.71 | (4.62) | -10.71 | (2.94) |
| SC | -4.95 | (5.04) | 2.34 | (2.58) | -5.79 | (2.67) | -3.73 | (4.67) |
| SD | -16.32 | (2.87) | -1.93 | (1.04) | -10.21 | (1.96) | -13.51 | (2.79) |
| TN | 4.93 | (11.81) | -2.92 | (2.54) | 7.19 | (10.26) | 9.27 | (13.97) |
| TX | 6.18 | (6.07) | 2.77 | (1.97) | 3.53 | (4.58) | 0.57 | (5.46) |
| UT | 1.17 | (11.91) | -1.25 | (2.21) | 3.31 | (12.27) | -1.46 | (10.79) |
| VA | -3.51 | (5.58) | 0.73 | (3.94) | -4.32 | (3.11) | 0.03 | (6.46) |
| WA | -2.93 | (5.64) | 0.84 | (2.27) | -2.07 | (5.04) | -4.55 | (5.26) |
| WI | 2.01 | (8.00) | 3.48 | (4.11) | -1.38 | (5.07) | -0.90 | (7.04) |
| WV | -3.05 | (4.71) | 0.29 | (2.50) | 0.24 | (4.36) | -2.40 | (4.72) |
| WY | -14.69 | (2.87) | -2.19 | (1.04) | -10.21 | (1.96) | -13.46 | (2.82) |
| States with < 2 teaching hospitals | 1.00 | (9.39) | 0.48 | (1.07) | -1.29 | (6.29) | 0.92 | (8.62) |
| Constant | 14.69 | (2.87) | 2.19 | (1.04) | 10.21 | (1.96) | 13.51 | (2.79) |
| Number of observations | 1110 | | | | | | | |

Notes: All regressions were performed on the subsample of teaching hospitals that received a cap decrease under Section 5503 and those that did receive any type of cap change.
